# Cholinergic activity is essential for maintaining the anterograde transport of Choline Acetyltransferase in ***Drosophila***

**DOI:** 10.1038/s41598-018-26176-z

**Published:** 2018-05-23

**Authors:** Swagata Dey, Krishanu Ray

**Affiliations:** Department of Biological Sciences, Tata Institute of Fundamental Research, Mumbai, India

## Abstract

Cholinergic activity is essential for cognitive functions and neuronal homeostasis. Choline Acetyltransferase (ChAT), a soluble protein that synthesizes acetylcholine at the presynaptic compartment, is transported in bulk in the axons by the heterotrimeric Kinesin-2 motor. Axonal transport of soluble proteins is described as a constitutive process assisted by occasional, non-specific interactions with moving vesicles and motor proteins. Here, we report that an increase in the influx of Kinesin-2 motor and association between ChAT and the motor during a specific developmental period enhances the axonal entry, as well as the anterograde flow of the protein, in the sensory neurons of intact *Drosophila* nervous system. Loss of cholinergic activity due to  Hemicholinium and Bungarotoxin treatments, respectively, disrupts the interaction between ChAT and Kinesin-2 in the axon, and the episodic enhancement of axonal influx of the protein. Altogether, these observations highlight a phenomenon of synaptic activity-dependent, feedback regulation of a soluble protein transport *in vivo*, which could potentially define the quantum of its pre-synaptic influx.

## Introduction

Synaptic activity is essential for the development and maintenance of neuronal circuits. It regulates the presynaptic influx of vesicles and organelles^1–4^. Several soluble proteins are selectively enriched in the axon^5,6^ and synapses^7–9^. Transport of these proteins plays a major role in both the assembly and maintenance of synaptic activity^10–12^. Also, the onset of several neuropathies is correlated to an abnormal transport of soluble proteins^13–17^. However, little is known about the regulation of their transport in the axon.

Currently, all soluble axonal transport phenomena are described as constitutive processes driven by either stochastic or non-specific interactions with motors or vesicular cargoes in the neighborhood^18–20^. In contrast, soluble forms of Dynein and ChAT are transported directly by Kinesin-1 and Kinesin-2, respectively, towards the synapse^20–22^. Although Dynein flux is constitutive, ChAT movement in axon acquires an anterograde bias contributed by the heterotrimeric Kinesin-2 during a certain developmental stage^22^, resulting in the pre-synaptic enrichment of the protein in the central nervous system of *Drosophila*^23^. The existing slow transport hypotheses, however, cannot explain the episodic movement and regulated pre-synaptic influx of soluble ChAT.

Acetylcholine (ACh) mediated neurotransmission is implicated in several cognitive functions^24–26^, and loss of cholinergic activity is indicated to cause dementia and neurodegenerative disorders. ACh is regenerated through the acetylation of choline by Choline acetyltransferase (ChAT), a soluble enzyme synthesized in the cell body and enriched at the presynaptic compartments^9^. Local recruitment of cholinergic machinery was found to promote neurite outgrowth and maintenance of motor activity in zebrafish larvae^27^. In *Drosophila*, a complete loss of zygotic ChAT function in the homozygous *cha* mutants caused progressive paralysis and lethality at nonpermissive temperatures^28,29^, and an increased presynaptic localization of ChAT is suggested to promote synapse assembly in the ventral ganglia of larval brain^30^. Interestingly, it also induced behavioral changes of *Drosophila* larvae, indicating a possible correlation between the altered transport and synaptic functioning.

Therefore, to understand the mechanism providing the anterograde bias to the bulk of ChAT movement and the impact of its presynaptic activity on the transport, we estimated interactions between ChAT and Kinesin-2 motor subunit *in situ* at different developmental stages. We also perturbed the cholinergic activity using pharmacological reagents and studied the effect on this transport. We used a high-sensitivity detector for data acquisition that enhanced the signal-to-noise ratio substantially as compared to the earlier report^22^. The results indicate that a temporally-restricted association with Kinesin-2, during 77–78 hours after egg laying (AEL), throughout the neuron drives the episodic movement of the bulk of ChAT towards synapse. A step increase in the axonal levels of the motor during 77–78 h AEL and cholinergic activity enhanced the entry and anterograde flux of ChAT in axons. The bulk movement of ChAT appears to evolve from a restricted to a directed, facilitated diffusion during this period.

## Results

### Kinesin-2 is required for the episodic accumulation and bulk anterograde movement of ChAT in *lch5* axons

**Figure 1:**
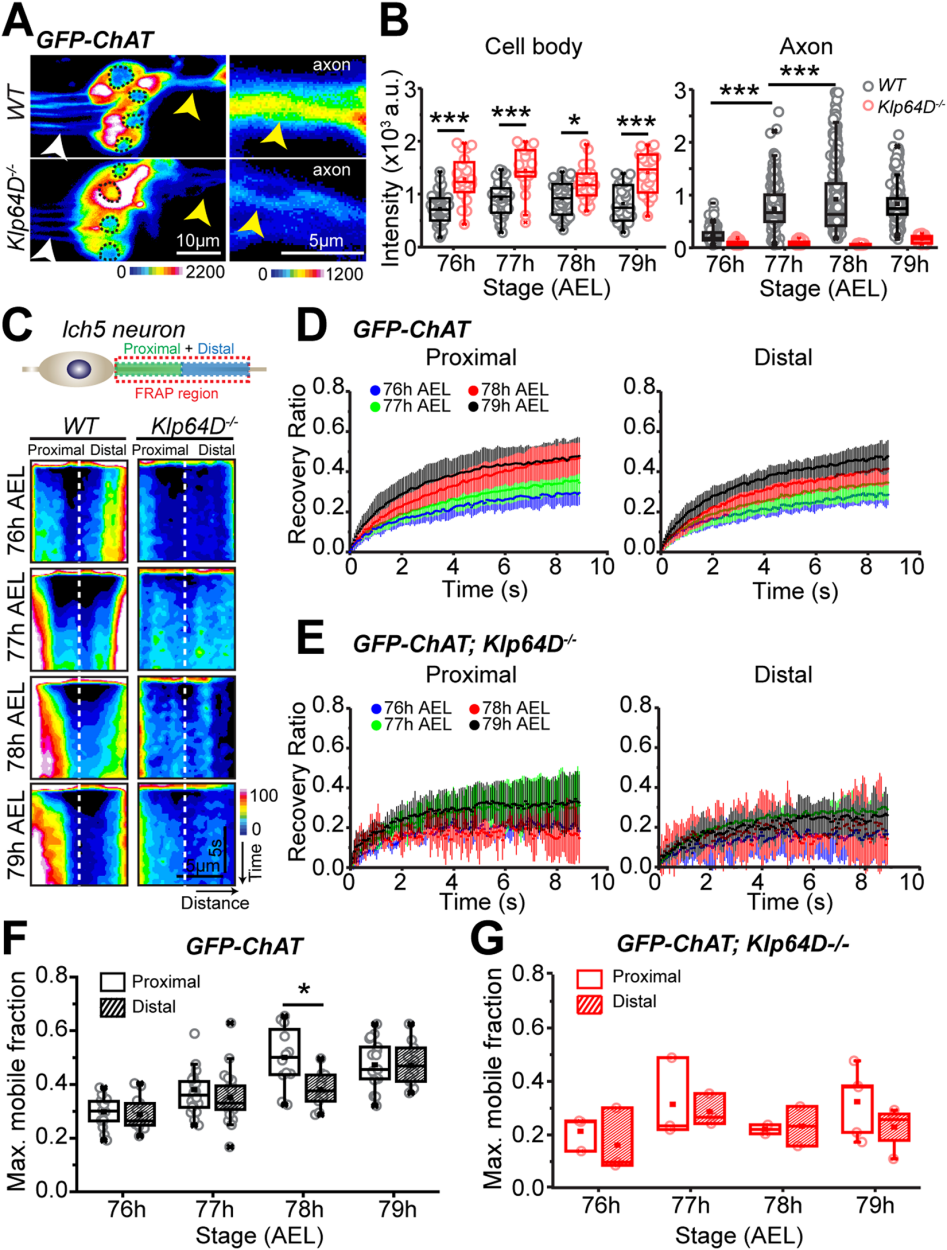
Role of Kinesin-2 in the episodic anterograde flow of ChAT in *lch5* axons. (**A**) Pseudocolored images depict the levels of GFP-ChAT fluorescence in different regions of the *lch5* neurons in the wild-type and *Klp64D*^*k1/A8*.*n123*^ (*Klp64D*^*−/−*^) mutant backgrounds. The axons (yellow arrowhead) and dendrites (white arrowhead) are indicated in each panel. The right panels indicated magnified views of the axonal bundles. (**B**) Quantification of GFP-ChAT fluorescence in the cell body and proximal axonal segments of lch5 neurons in the wild-type and *Klp64D*^*k1/A8*.*n123*^ (*Klp64D*^*−/−*^) mutant larvae. (**C**) Schematic showing the relative position of the axon segment of a *lch5* neuron used for the FRAP assay, and kymographs depict the FRAP profiles of GFP-ChAT measured from the 10 µm axon segment during 76–79 h AEL in the wild-type and *Klp64D*^*−/−*^ backgrounds. (**D**,**E**) The average (±S.D.) recovery ratios of GFP-ChAT fluorescence (relative recovery) at the proximal and distal segments of the photobleached axonal region in wild-type (**D**) and *Klp64D*^*−/−*^ (**E**) backgrounds (N ≥ 5). (**F**,**G**) Box and scatter plots depict maximum mobile fractions estimated from the FRAP profiles of the proximal and distal segments of wild-type (**F**) and *Klp64D*^*−/−*^ (**G**) neurons expressing GFP-ChAT. The pair-wise significance of differences was estimated using one-way ANOVA, and the p-values < 0.05 (*), and < 0.001 (***) are indicated on the panels.

To define the bulk movement characteristics of ChAT and assess the contribution of Kinesin-2 in the process in a mature neuron, we investigated the accumulation of GFP-ChAT in wild-type control, *Klp64D*^*k1/A8*.*n123*^ (*Klp64D*^*−/−*^) hemizygous, and *Klp64D*^*k5*^ homozygous mutant backgrounds in *lch5* axons during 76–79 h AEL. Expression of *chaGal4* > *GFP-ChAT* in the homozygous *cha*^*ts2*^ background rescued the high-temperature paralysis observed at 32 °C (Supp. Movie S1). Estimation from the 10 µm long region of axons next to the cell bodies of *lch5* neurons revealed a characteristic increase in the integral GFP-ChAT fluorescence during 76–78 h AEL in wild-type larvae (Fig. 1A,B). GFP-ChAT fluorescence intensity in the axons was reduced to near zero level at all developmental stages in the *Klp64D*^k5^ *and Klp64D*^−/−^ backgrounds (Figs 1A,B and S1). The defect was rescued by the expression of UAS-KLP64D-TEVHis transgene in the homozygous *Klp64D*^*k5*^ mutant background (Figure S1). Consistent with the observations made in the medial interneuron^22^, the Fluorescence-Recovery-After-Photobleaching (FRAP) kymographs of GFP-ChAT obtained from the wild-type background revealed a progressive increase in the GFP-ChAT flow into the proximal segment during 76–78 h AEL (Fig. 1C, Supp. Movie S2), which was absent in the *Klp64D*^−/−^ background (Fig. 1D,E). Together these observations suggested that bulk localization of ChAT in the axon is intrinsically episodic and Kinesin-2 is necessary for its axon entry.

Differential of the net anterograde and retrograde flow within an axon would determine the enrichment of a particular component at the synapse. Both the proximal and distal recovery ratios of GFP-ChAT progressively increased during 76–79 h AEL (Fig. 1D). This temporal modulation of GFP-ChAT flow was absent In the *Klp64D*^*−/−*^ background (Fig. 1E). The proximal and distal mobile fractions in control were comparable for most of the observed period (Fig. 1F), except at 78 h AEL, when the maximum mobile fraction was marginally higher in the proximal half (0.5 ± 0.1) than in the distal half (0.41 ± 0.1) (Fig. 1F). Thus, it generated a net anterograde ChAT flux during this period. Although in the Kinesin-2 mutant, the relative recovery (0.29 ± 0.07) was similar to that of wild-type neurons at 76 h AEL (0.28 ± 0.03) (Fig. 1G), average mobile fractions in the proximal (0.26 ± 0.05) and distal (0.22 ± 0.05) segments were significantly reduced in subsequent stages (Fig. 1G). Further, the anterograde bias observed in wild-type neurons was abolished in the mutant background. These results elucidated two important roles of Kinesin-2 during axonal transport of ChAT: the axonal entry and anterograde progression of the molecule. The axonal flow of GFP was uninterrupted in Kinesin-2 mutants^22^, suggesting that loss of Kinesin-2 specifically disrupted the flow of ChAT without altering the structure of the axon significantly.

### An episodic Kinesin-2 influx, modulated by the ‘tail’ domain of the 2α subunit, coincides with that of the bulk ChAT movement

In *Drosophila*, a functional Kinesin-2 motor comprises of the motor subunits KLP64D (2α), KLP68D (2β), and the accessory protein DmKAP^31^. Previous studies suggested that KLP64D is unstable as a monomer *in vitro*^32^. However, overexpression of the transgenic KLP64D-GFP and KLP68D-YFP through the ectopic *chaGal4* driver could result in persistence of the soluble monomers *in vivo*. Therefore, to monitor the correlation between the ChAT movement with that of the active motor complex, we monitored the FRAP profiles of KLP64D-GFP and KLP68D-YFP during 76–79 h AEL in two independent set of experiments.

**Figure 2:**
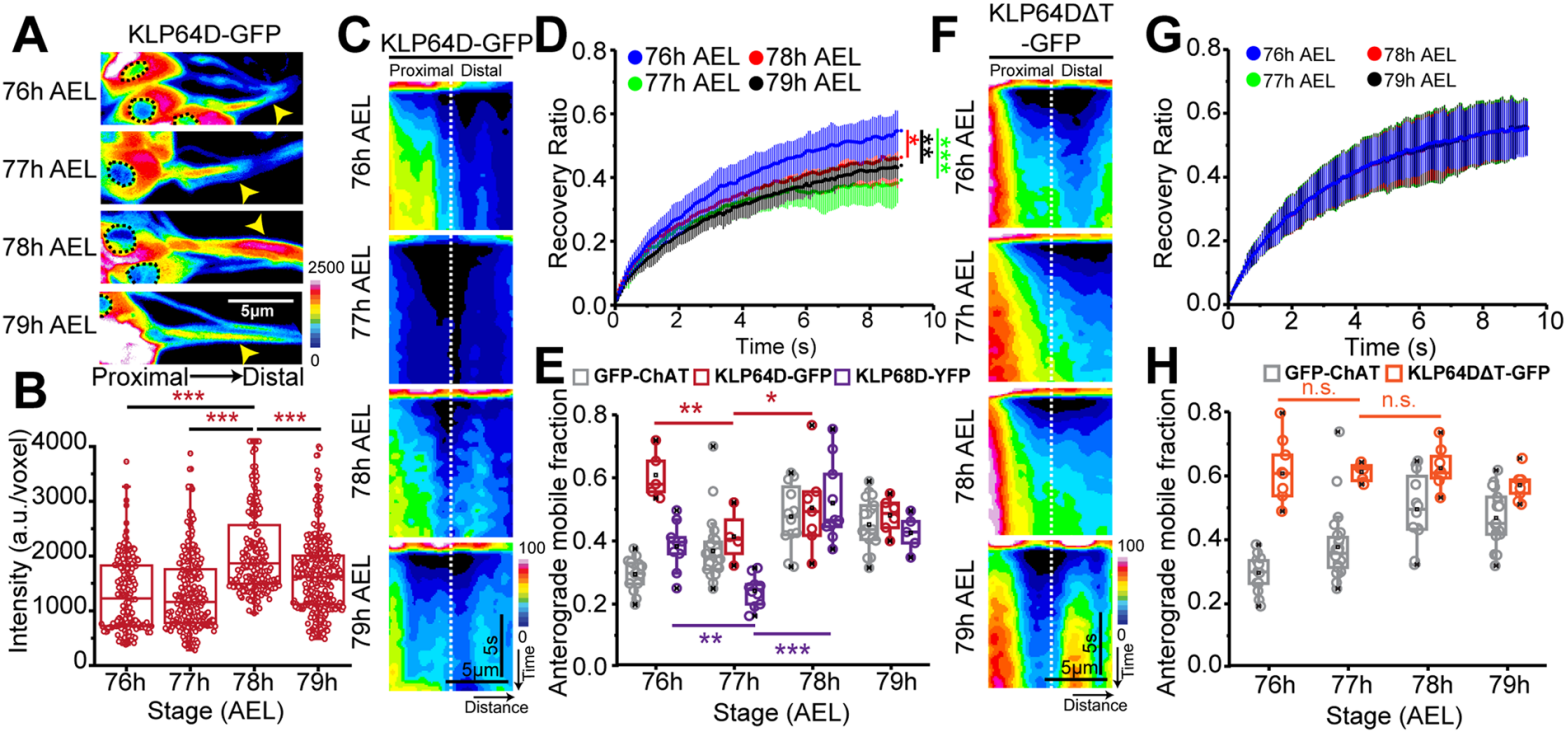
Flow characteristics of Kinesin-2 motor subunits in *lch5* axons. (**A**,**B**) Pseudocolored intensity-heat-map images (A), average fluorescence intensities (**B**) of KLP64D-GFP depict the relative distribution of the recombinant motor in the *lch5* neurons. Black dotted perimeter outlines nuclei in each neuron, and yellow arrowheads show the axons. Intensities in the 10 μm proximal segment of *lch5* axons were estimated at different stages of development. (**C**) KLP64D-GFP FRAP kymographs, obtained during 76–79 h AEL from the 10 µm axon segment of *lch5* neurons (schematic, Fig. 1C), and presented in the pseudo-colored intensity-heat-map. (**D**) Relative recovery profiles of KLP64D-GFP FRAP during 76–79 h AEL (N ≥ 6). (**E**) The maximum anterograde mobile fraction obtained from the single exponential fit of the FRAP profiles of GFP-ChAT (gray), and KLP64D-GFP (red), and KLP68D-YFP (purple) in the proximal axon segments of *lch5* neurons. (**F**) KLP64DΔT-GFP FRAP kymographs depict relative recovery of fluorescence during 76–79 h AEL in the 10 µm axonal segments of *lch5* neurons. (**G**,**H**) Relative recovery profiles of KLP64DΔT-GFP FRAP during 76–79 h AEL (**G**), and maximum anterograde mobile fractions obtained from the single exponential fits of the FRAP profiles of KLP64DΔT-GFP (orange) and GFP-ChAT (gray)(**H**). The pairwise significance of differences was estimated using one-way ANOVA and the p-values (ns, * < 0.05, ** < 0.01, *** < 0.001) are indicated on the panel.

We observed a marked increase in the levels of KLP64D-GFP between 77 and 78 h AEL, which dropped subsequently at 79 h AEL (Fig. 2A,B). Similar observation was made using KLP68D-YFP (Figure S2A,B). Comparatively higher expression levels of the *chaGal4* driver at 77–78 h AEL, or, a transitory increase in the association between the motor and cargo, could generate the episodic influx of GFP-ChAT, KLP64D-GFP, and KLP68D-YFP. Kinesin-2 is a heterotrimeric complex, and a previous study suggested that KLP64D can only remain stable in a heterodimer with KLP68D^32^. Hence, the axonal influx of the KLP64D-GFP may also indicate a higher dynamic movement of Kinesin-2 into the axon. FRAP estimation of the mobile fraction of the tagged motor subunits in the axon elicited different patterns of KLP64D-GFP and KLP68D-YFP recovery, distinct from that of GFP-ChAT. KLP64D-GFP flow in the proximal segment was highest at 76 h AEL, which dipped significantly at 77 h AEL, and increased in the subsequent stages (Fig. 2C,D, Supp. Movie S3). A similar dip was noticed in the KLP68D-YFP FRAP profiles (Figure S2A–C). Inexplicably though, we noticed that the highest KLP68D-YFP recovery occurred at 78 h AEL. As all these recombinant proteins were expressed by *chaGal4*, it ruled out the possibility that the expression levels could drive the axonal influx, and further suggested that the motor influx into axon may occur independent of ChAT, especially at 76 h AEL.

The C-terminal tail domain of the Kinesin-2α motor subunit, KLP64D, interacts with ChAT and is essential for its axonal flow^22^. We reasoned that the anomalous dip in the FRAP values of the motor subunits at 77 h AEL (Fig. 2E) could result from a temporal block in the motor activity, or retardation by *en masse* binding with some soluble cargo like ChAT. We examined the kinetic properties of the motor which appeared uniform throughout development. The recovery fronts of KLP64D-GFP and KLP68D-YFP progressed at comparable rates in both the anterograde (Front Velocity − 1.23 ± 0.25 µm/s, Displacement − 1.97 ± 0.2 µm) and retrograde (Front Velocity − 1.09 ± 0.2 µm/s, Displacement − 1.89 ± 0.4 µm) directions at all stages (Figure S2D,E). Therefore, to test this possibility, we estimated the anterograde flow of KLP64DΔT-GFP, which is unlikely to bind ChAT^22^. Expectedly, KLP64DΔT-GFP FRAP did not have a dip at 77 h AEL (Fig. 2F). Analysis of the recovery curves also revealed a uniform flow of KLP64DΔT-GFP throughout the larval stages (Fig. 2G), which was significantly higher than that of the GFP-ChAT (Fig. 2H). These observations suggested that interaction between Kinesin-2 and soluble ChAT could alter the bulk flow characteristics of the motor at 77 h AEL. However, it did not explain the subsequent appreciation of the recovery rates of both the motor subunits and the cargo.

### Increased interaction between Kinesin-2 and ChAT in *lch5* axons overlaps with the peak anterograde movement of the bulk of ChAT

**Figure 3:**
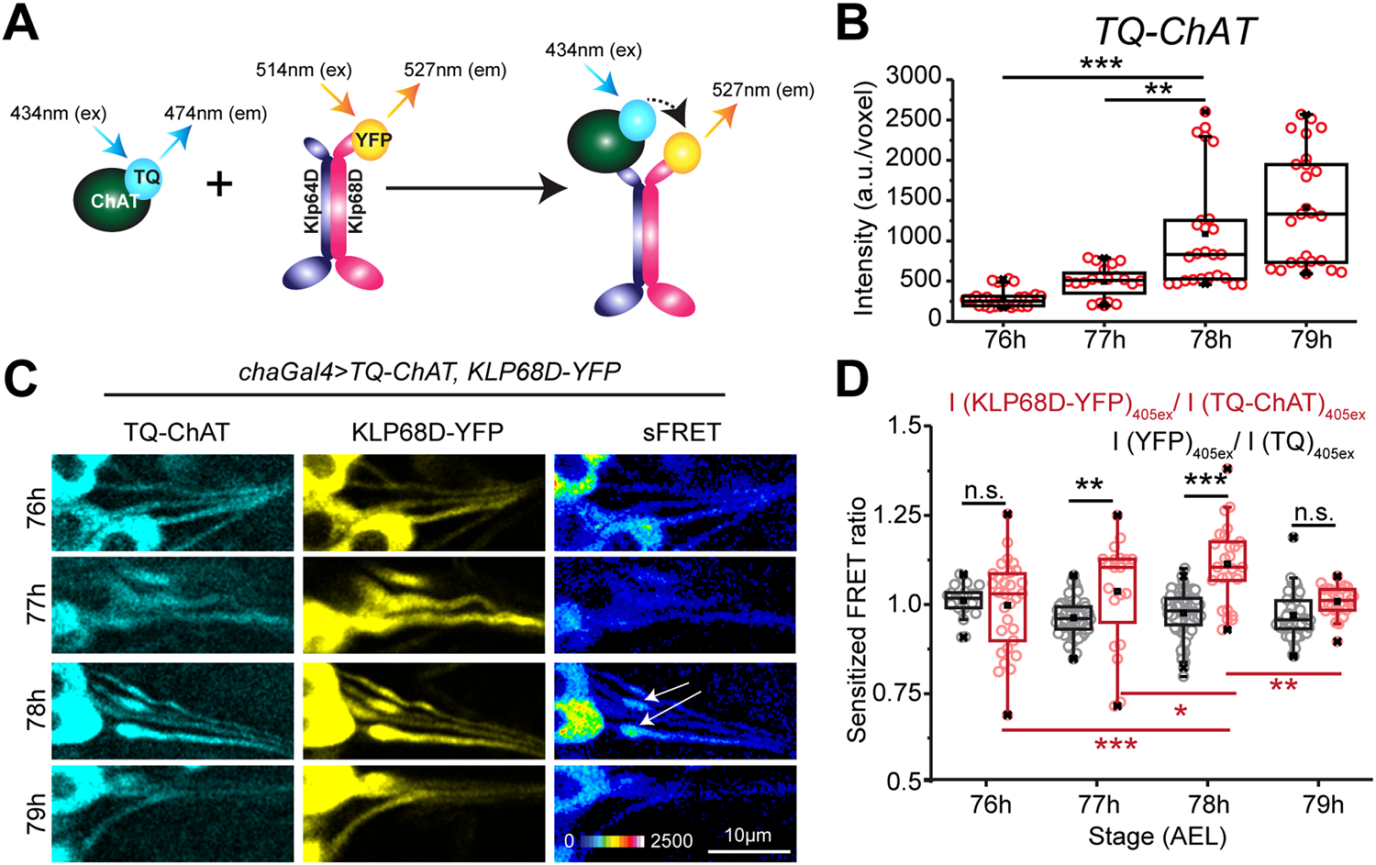
An estimate of the dynamic association between ChAT and Kinesin-2 in *lch5* axons during transport at different developmental stages. (**A**) Schematic illustrates the sFRET assay principle adopted to measure dynamic interactions between TQ-ChAT and KLP68D-YFP subunit of Kinesin-2. (**B**) TQ-ChAT accumulation in the *lch5* axon segment during 76–79 h AEL. (**C**) Raw images (TQ, YFP, and sFRET channels) obtained from the *lch5* neurons expressing TQ-ChAT/KLP68D-YFP and TQ/sYFP (control) pairs respectively during 76–79 h AEL. Note the increase observed in the FRET channel at 78 h AEL (white arrows). (**D**) The combined box and scatter plots depict sFRET ratios in the axon segments obtained from the TQ-ChAT/KLP68D-YFP and TQ/sYFP backgrounds during 76–79 h AEL. The pairwise significance of differences was estimated using one-way ANOVA and the p-values (ns, * < 0.05, ** < 0.01, *** < 0.001) are indicated on the panel.

To identify the temporal regime of interaction between Kinesin-2 motor subunits and ChAT during the transport in *lch5* neurons, we used the Forster’s Resonance Energy Transfer (FRET) assay. A recombinant ChAT tagged with the mTurquoise fluorescent protein (TQ, Ex 434 nm, Em 474 nm)^33^, along with the KLP68D-YFP, was expressed using *chaGal4*. Because the ChAT binding region in the KLP64D tail is unknown, we reasoned that the addition of a fluorescent protein tag at C-terminus of KLP64D might hinder the interaction *in situ*. Hence, KLP68D-YFP was chosen as the acceptor partner in the FRET assay. Sensitized FRET (sFRET) emissions from the TQ-ChAT and KLP68D-YFP pair was assessed during 76–79 h AEL (Fig. 3A). The sFRET values between untagged, soluble forms of TQ and sYFP were used as negative control (Fig. 3A). Like the GFP-ChAT, TQ-ChAT also progressively accumulated in the *lch5* axon segment during 76–78 h AEL (Fig. 3B), and the sFRET ratios of KLP68D-YFP and TQ-ChAT emissions in the compartment gradually increased from 76 h AEL onwards until 78 h AEL (Fig. 3D). In comparison, the emissions of soluble TQ and sYFP were maintained at a level comparable to that of the highest levels of TQ-ChAT and KLP68D-YFP throughout 76–79 h AEL (Figure S3). The sFRET between TQ and sYFP yielded the estimates of non-specific interaction due to crowding in the *lch5* axon segment, which was consistently lower and comparable to the 76 h sFRET between TQ-ChAT and KLP68D-YFP (Figure S3). Therefore, we conjectured that a higher fraction of ChAT associated with a relatively higher fraction of Kinesin-2 motor resulting in its enhanced anterograde flux, and thereby, increasing the bulk accumulation in axon at 78 h AEL.

To verify this idea, we carried out extended sFRET measurements at 5 frames per second for up to 40 seconds at each stage during 76–79 h AEL. It revealed a progressive decline in the TQ-ChAT emission concomitant to a proportional increment in the interacting KLP68D-YFP (FRET channel) emission at 78 h AEL, causing a net increase in the sFRET values (Figure S4, Supp. Movie S4). Therefore, we concluded that a progressively increasing association between a relatively larger amount of ChAT with Kinesin-2 at 78 h AEL could generate the net anterograde bias to the bulk ChAT movement at this stage. Also, the absence of obvious particulate characteristics in the sFRET kymographs suggested that the motor would associate with individual ChAT molecules instead of soluble aggregates of the protein, which is consistent with the density sedimentation data presented before^22^.

### Cholinergic activity regulates the ChAT influx and its interaction with Kinesin-2

Axonal transport of mitochondria^4^, vesicular cargoes^1^ and RNA-Protein complexes in dendrites^34^ are directly linked to synaptic function, and reduced synaptic activity promoted the retrograde transport^2^. To probe whether the synaptic activity of *lch5* neurons and activation of the post synaptic neurons could influence the episodic interaction between ChAT and Kinesin-2, we treated the larval preparations  with Hemicholinium (HC3) and Bungarotoxin (BTX), respectively^35–39^. HC3 treatment blocks choline reuptake by Choline Transporter (ChT) at the presynaptic membrane, thereby restricting ACh synthesis at the presynaptic terminal^35^. BTX inhibits the nicotinic Acetylcholine Receptor (nAChR) on the postsynaptic membrane, blocking the activation of the postsynaptic neurons in a cholinergic network^36–39^.

**Figure 4:**
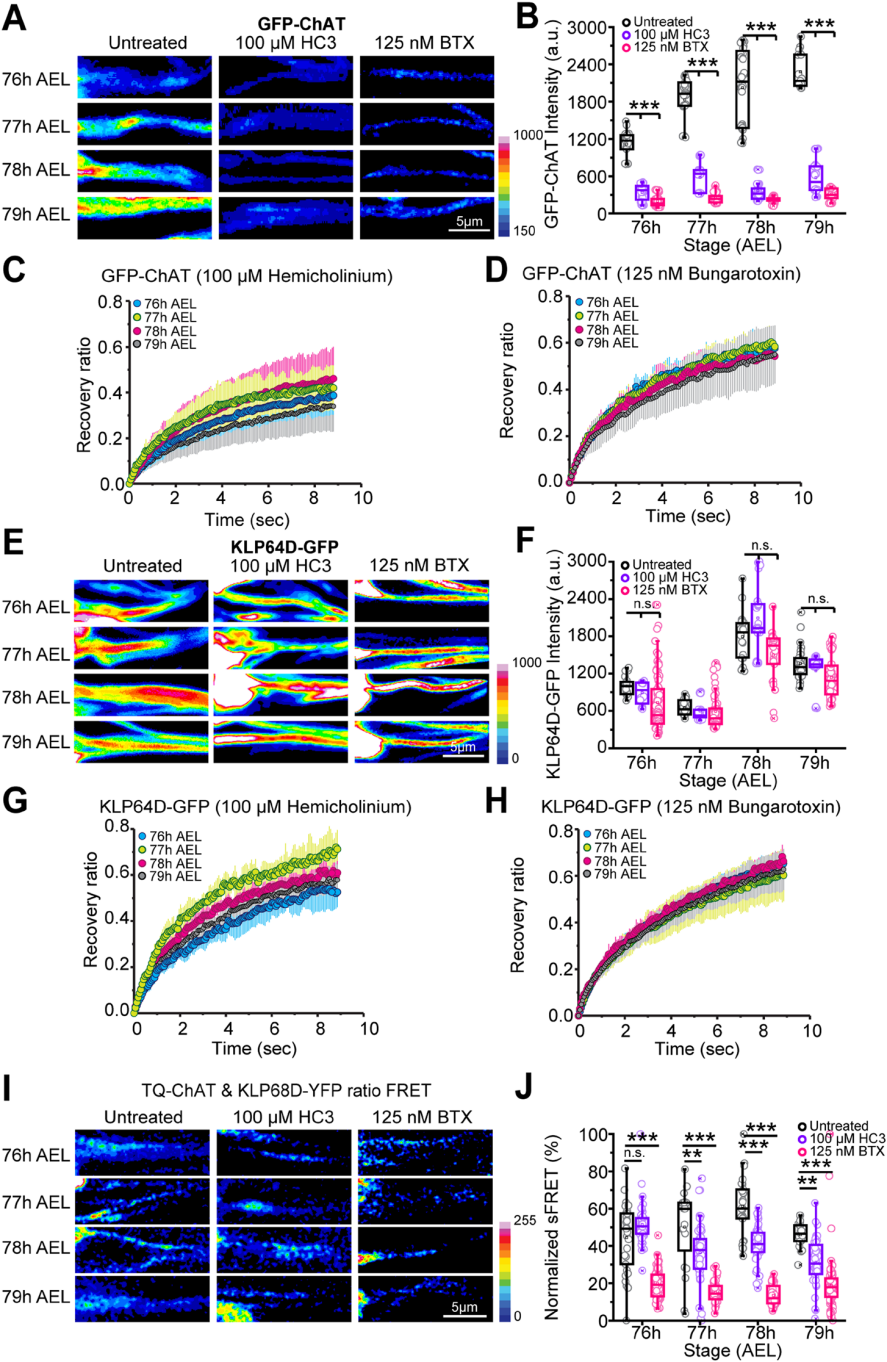
Effects of Hemicholinium (HC3) and Bungarotoxin (BTX) treatments on the axonal transport of ChAT and its association with the Kinesin-2 motor. (**A**,**B**) GFP-ChAT accumulation during 76–79 h AEL in the *lch5* axon segments of control, HC3, and BTX -treated preparations. The images are presented as pseudo-colored intensity-heat-map (**A**), and the fluorescence intensities (**B**) are presented as per Fig. 1B. (**C**,**D**) The relative recovery profiles of GFP-ChAT in the *lch5* axon segments upon treatment with Hemicholinium (**C**) and Bungarotoxin (**D**) estimated during 76–79 h AEL. (**E**,**F**) KLP64D-GFP accumulation in the *lch5* axon segments in control, HC3 and BTX-treated preparations during 76–79 h AEL. The images are presented as pseudo-colored intensity-heat-map (**E**), and the average fluorescence intensity (**F**) are plotted as Fig. 2B. (**G**,**H**) KLP64D-GFP recovery profile in the *lch5* axons treated with Hemicholinium (**G**) and Bungarotoxin (**H**) estimated during 76–79 h AEL. (**I**) sFRET distribution depicted in pseudocolor intensity-heat-map between TQ-ChAT and KLP68D-YFP in control, HC3, and BTX treated axon segments during 76–79 h AEL. (**H**) Normalized sFRET (%) between TQ-ChAT and KLP68D-YFP pair in control, HC3, and BTX treated axons. The values normalized across 76–79 h AEL under each condition. The pair-wise significance of differences assessed using one way ANOVA, and appropriate p-values (ns, * < 0.05, ** < 0.01, *** < 0.001) are indicated on figure panels.

Incubations in the buffer containing 100 µM HC3 for 20 min induced a visible reduction of the GFP-ChAT accumulation in axon (Fig. 4A). Total GFP-ChAT levels in the axon were diminished by 4–7.5 folds as compared to the controls during 76–79 h AEL (Fig. 4B), the FRAP kymographs revealed bidirectional recovery fronts (Figure S5A), and the GFP-ChAT mobility became uniform throughout the 76–79 h AEL period (Fig. 4C, Supp. Movie S5). Analysis of the maximum recovery in the proximal and distal compartments (Figure S5C) further indicated that the HC3 treatment abrogated both the axonal entry, as well as the anterograde bias in the GFP-ChAT flow. However, an average relative recovery of 41.5% during 76–79 h AEL, suggests that HC3 treatment hinders the active movement of GFP-ChAT without affecting its diffusional mobility. A similar, and rather severe, defect was observed in the presence of 125 nM BTX (Figs 4B, S5A,C). These treatments also eliminated the distinct temporal pattern of the FRAP recovery profile of GFP-ChAT observed during 76–79 h AEL (Fig. 4C and D, Supp. Movie S6).

In comparison, the KLP64D-GFP levels remained unaltered after the treatment (Fig. 4E–H); however, the characteristic dip in the KLP64D-GFP FRAP at 77 h AEL was abolished (Fig. 4G,H). No particular bias in the mobility of KLP64D-GFP was observed during 76–79 h AEL upon both the HC3 and BTX treatments (Figure S5B,D). These observations suggested that the loss of cholinergic activity could affect the interaction between Kinesin-2 and ChAT, reducing the axonal influx and loss of the anterograde bias in the flux of ChAT at 78 h AEL. To further test this hypothesis, we estimated sFRET between TQ-ChAT and KLP68D-YFP in the *lch5* axonal segments in the presence of HC3 and BTX (Fig. 4I). Although the relative sFRET values were not significantly altered at 76 h AEL, the treatment abolished subsequent increment in the % sFRET during 77–79 h AEL (Fig. 4J). Together, these observations indicated that cholinergic activation of the postsynaptic neurons is essential for promoting interactions between Kinesin-2 and ChAT in axons of presynaptic neurons.

## Discussion

Axonal transport of ChAT has been extensively studied in various organisms and neuron types^22,40–43^. Estimates of accumulated ChAT activity at the ligature of rat sciatic nerve suggested that the enzyme flows anterogradely at an average rate of ~1.2 mm/day^43^. Using the high-resolution FRAP we estimated a much faster max flow rate (1.8 µm/s or ~155 mm/day) in the axons of intact *lch5* neurons of *Drosophila* larvae, as compared to an earlier estimate (0.97 µm/s or ~83 mm/day) obtained from the short interneurons of the ventral ganglion^22^. A similar disparity in rates was reported for the other slow axonal cargoes such as neurofilaments, CaMKII, Synapsin, and Actin^19,44–47^. This apparent discrepancy in the rate estimates is a consequence of spatiotemporal characteristics of the transport which are reflected in the assaying paradigms and acquisition parameters. Besides the 76–79 h AEL interval in the third instar stage, we observed another episode of ChAT influx in *lch5* axons during 52–56 h AEL in the second instar stage (Dey S., *unpublished observation*). Assuming that ChAT transport episode is restricted to an hourly interval during each molt, the effective flow rate during a 24 h molting period would be 3.5 mm/day which correlates well to transport characteristics of ChAT as a slow rate component. These results are obtained from fully ensheathed functional neurons connected to the native circuitry at different developmental stages. Thus, it also provided near endogenous characteristics of the transport. With the improved observation capability, we found that the temporal parameters of the ChAT transport are consistent in both the small interneurons of ventral ganglion, as well as in the mature *lch5* neurons, suggesting that the episodic nature of the ChAT transport is an intrinsic property.

ChAT was reported to bind directly to the C–terminal tail domain of the Kinesin-2α subunit *in vitro*^22^. Kinesin-2 is essential for two distinct aspects of the transport process – entry into the axons and for conferring the anterograde bias observed at 78 h AEL. The FRAP and FRET assay further suggested that the episodic movement of the bulk of ChAT is initiated through a transient association with the Kinesin-2 motor throughout the neuron. Although the motor was present in the axon all throughout, the association was limited to an hourly interval or less during late larval development. Studies showed that temporal switching of association from Kinesin-3 (Unc-104) to Dynein shifts the transport of Rab3 vesicles from anterograde to retrograde in the DD neurons of *C*. *elegans*^48^. This change in the modality of Rab3 vesicle transport in the DD neurons was associated with synapse restructuring induced by cyclin, CYY-1 and the cyclin-dependent kinase, CDK5^48^. The ChAT transport episodes appear to closely follow the molting cycle, which is induced through the surge of Juvenile Hormone (20-Hydroxyecdysterone) in *Drosophila* larvae^49^. Considering the timescale of the transport modulation, certain post-translational modifications such as phosphorylation could enhance the affinity between ChAT and Kinesin-2. For example, Calmodulin Kinase II (CaMKII)-mediated phosphorylation of Kinesin-2 tail is suggested to increased the transport of N-Cadherin towards the synapses^50^. A preliminary motif scan also revealed putative Casein Kinase II-mediated phosphorylation sites in ChAT and the tail domains of Kinesin-2. Therefore, certain developmental signaling cues could trigger the episodic interaction between ChAT and Kinesin-2 through phosphorylation or other post-translational modifications.

**Figure 5:**
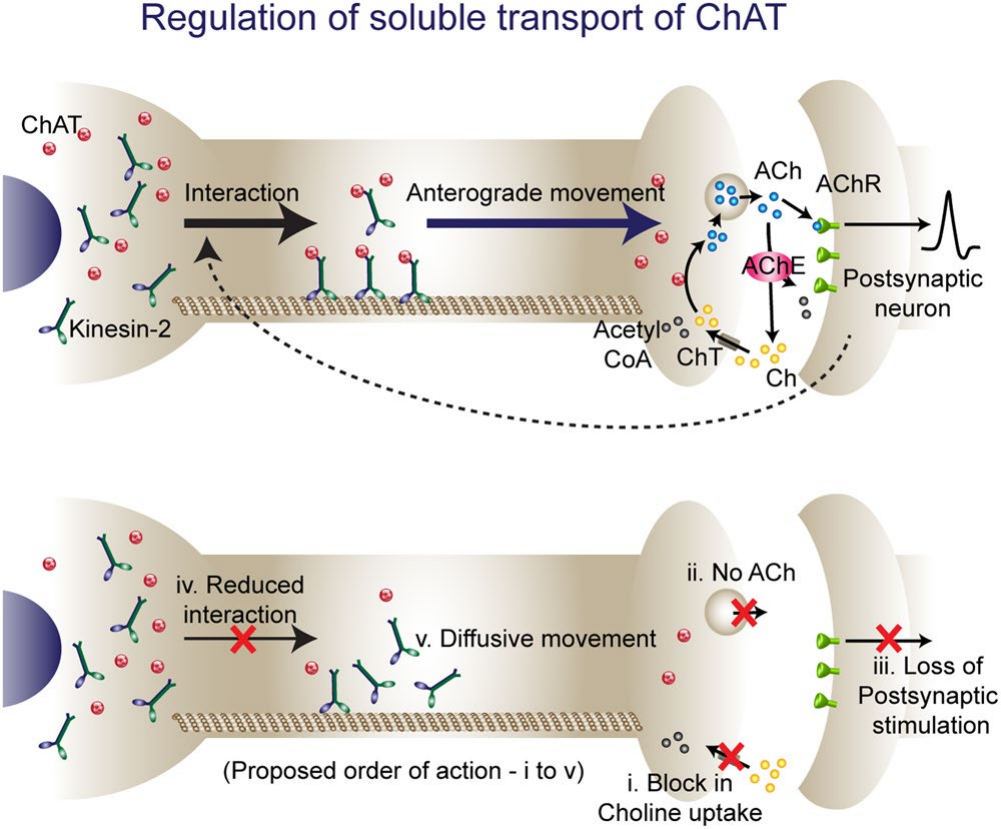
Synaptic activity regulates the anterograde soluble transport of ChAT. Schematic showing possible role of a retrograde signaling feedback that regulates the axonal transport of ChAT. The interaction between ChAT and Kinesin-2 is required for the axonal entry of ChAT and its anterograde propagation. Block in the choline uptake at the presynaptic terminal causes loss in acetylcholine synthesis and release. Lack of postsynaptic stimulation might elicit changes in the presynaptic neuron which further impinge on the interaction between Kinesin-2 and ChAT. As a result ChAT entry into the axon and its active transport by Kinesin-2 are impaired.

Kinesin-2 plays an essential role in the anterograde movement of ChAT in *Drosophila* and mouse axons^22,23,51,52^. Apart from ChAT, both Rab4, and Acetylcholinesterase (AChE) are transported by Kinesin-2 in axons^30,53^, and functions of these three proteins are implicated in synapse homeostasis^30,54^. Above data indicates that direct interaction between the motor and ChAT for brief duration induced the episodic flow towards synapse, and synaptic activity is essential for this interaction (Fig. 5). Blocking the acetylcholine synthesis through the HC3 treatment, and activation of the post synaptic neurons through the inhibition of actylcholine receptors by BTX, respectively, was found to reduce the axonal entry and disrupt the episodic nature of ChAT transport within a short time, indicating that synaptic activity regulates axonal entry through a fast retrograde communication. Neuronal depolarization and synaptic activity have also been suggested to regulate the axonal transport either directly or indirectly, for example, the activity-dependent synaptic capture of Dense Core Vesicles, the depolarization-triggered redistribution of κ-opioid receptor mRNA, and effect on mitochondrial trafficking due to activity-induced changes in the intracellular calcium and ATP^3,4,55^. In the context of axonal injury, the earliest communication between the distal end of the axon and the soma is established by ionic fluxes^56^. Such an event is conjectured to modulate the signaling molecules and transcriptional profile of the neuron, activate redistribution of mRNA and nuclear proteins, and alter the structural characteristics of the AIS via retrograde signaling complex^57–60^. Thus, continued stimulation of *lch5* neurons during larval stages^61^ together with certain post-translational modifications induced by the developmental cues could define the episodic postsynaptic feedback and interactions between ChAT and Kinesin-2. Subsequently, the enhanced transport could enhance the synaptic contacts in the ventral ganglion.

## Methods

### *Drosophila* culture and stocks

All the fly stocks (Table S1) were reared on standard cornmeal agar medium at 25 °C with 12-hour light-dark cycle. All experiments were done on dissected third instar larvae with the intact nervous system as described before^30^.

### Cloning and transgenic generation

mTurquoise (TQ), a variant of Cyan Fluorescent Protein^33^, was subcloned into the EcoR1/Not1 sites of the pUAST vector containing a ChAT-ORF inserted in the Not1/Kpn1 site of the same MCS. The 748 bp TQ-ORF amplicon obtained using appropriate primers was digested with EcoR1 and Not1, and the gel-eluted band was ligated with the recipient pUAST-ChAT plasmid^22^. The transgenic fly stocks were generated at the C-CAMP facility of NCBS, TIFR, Bangalore. A similar strategy was used to clone the ORFs of TQ and SYFP in the pUAST vector.

### *Drosophila* sample preparation

Third instar larvae were dissected in Jan and Jan Buffer (pH = 7.2) containing 12.8 mM NaCl, 0.2 mM KCl, 0.4 mM MgCl_2_, 0.18 mM CaCl_2_, 3.53 mM Sucrose and 0.5 mM HEPES, through a dorsal incision along the posterior to anterior axis. After removal of internal organs, the prep was mounted in the same buffer with cuticle side facing towards the coverslip for imaging of the *lateral chordotonal* (*lch5*) neurons.

### Image acquisition

Lateral chordotonal neurons were imaged using 60×/1.4NA objective at a scaling of 0.115 µm × 0.115 µm on the Olympus FV1000SPD laser scanning confocal microscope at a frame rate of 10 Hertz for 10 seconds. For this study, the images were acquired from the proximal, 20 µm segment of the *lch5* axons. The FRAP assay was limited to an area of 10.35 × 2.3 µm (90 × 20 pixels) adjacent to the cell body. It was first imaged for 0.5 sec (5 frames) at an optimal laser power, then photobleached for 1.0 sec (10 frames) at 1.1 mW, and subsequently, the time-lapse images were acquired for the defined durations using the optimized laser power. The PMT gain was maintained at an optimum level for all measurements.

Sensitized Forster’s Resonance Energy Transfer (sFRET) measurements were performed on neurons expressing TQ-ChAT and KLP68D-YFP. The tissue was illuminated with 405 nm and 514 nm for the donor and the acceptor excitations, and the emissions were recorded using the secondary band pass filters, 480–495 nm (TQ channel), and 535–565 nm (FRET channel), respectively, using the high sensitivity detectors (HSD). To obtain spatiotemporal information of the FRET, time-lapse images were acquired in a region [40.595 µm (353 px) × 11.5 µm (100 px)] covering the relevant portion of the tissue at a frame rate of 5 Hz.

### Quantification and Analysis

The images and videos were quantified using ImageJ^®^. All the images were background corrected and normalized to the maximum. Integrated intensities obtained from the FRAP region were normalized to pre-bleach and post-bleach intensities and plotted against time. The FRAP curves were fitted to the following single exponential equation:

$${I}_{t}(t)={I}_{max}(1-{e}^{-\tau \ast t})$$

The maximum recovery amplitude (*I*_*max*_) and the half-time (*t*_*1/2 = *_*ln0*.*5/−τ*) parameters were obtained from the fit. The vectorial bias of the bulk flow along the proximo-distal axis of the axons was inferred from the values obtained from the proximal and distal compartment each measuring 5.18 × 1.15 µm^2^. The movement of the bulk over time and across the photobleached segment was observed as a front in the kymographs obtained from the FRAP movies. A Gaussian filter was applied to achieve a smooth bulk front in the kymographs. To estimate front velocity and displacement, the kymographs with raw intensities were converted to a ratio kymograph where pixel intensity could range from 0 to 1. The interface of FRAP pixels with 0 value and recovered pixels with values >0 was manually traced to estimate the front velocity and displacement in each kymograph. Depending upon the angle of the trace, the direction (anterograde and retrograde) was assigned to the front.

For the sFRET quantification, integrated intensities from the region measuring 10.35 × 1.15 µm^2^ at proximal axon in the neuron were obtained. sFRET values were represented as a ratio of the FRET channel (KLP68D-YFP or YFP intensity with 405 excitations) to the TQ channel (TQ-ChAT or TQ with 405 excitations).

$$r=\frac{I{(KLP68D-YFPorYFP)}_{405ex}}{I{(TQ-ChATorTQ)}_{405ex}}$$

Temporal measurements of sFRET were carried out by measuring the mean intensities of a line profile on the axons in the FRET and TQ channel. The FRET images or kymographs were normalized using the mean intensity of the TQ channel to obtain the sFRET images. Normalized sFRET (%) of TQ-ChAT and KLP68D-YFP pair were obtained by normalization across 76–79 h AEL in each condition.

$$ \% r=\frac{r-{r}_{min}}{{r}_{max}-{r}_{min}}\times 100$$

All the statistical comparisons were carried out using ANOVA and p-values were calculated according to Bonferroni Test of corrected measures in Origin®. p-values for the respective datasets are indicated on the panels.

### Treatment with Hemicholinium (HC3) and Bungarotoxin (BTX)

HC3 is a drug that blocks the reuptake of Choline in the presynaptic terminal by inhibiting the ChT channel^35^ and BTX inhibits nAChR on the postsynaptic membrane^36–38^. *Drosophila* third instar larvae of appropriate age (76–79 h AEL) were dissected as fillet preps in the Jan and Jan buffer containing 100 µM HC3 or 125 nM BTX and mounted in the same buffer. The fillet preps were exposed to HC3 or BTX for a total duration of 20 min including the dissection and imaging.

### Behavioral assay

*cha*^*ts2*^ and the rescue flies were reared at 18 °C till eclosion. Following eclosion, the adult flies were kept at 32 °C for 24 hours and assessed for paralysis. Paralysis was checked using the climbing assay where flies were put in a vertical glass tube and monitored for a duration of a minute.

## Electronic supplementary material

Supplemental Figures and Legends

Supplemental Video S1

Supplemental Video S2 (related to Figure 1):

Supplemental Video S3 (related to Figure 2):

Supplemental Video S4 (related to Figure 3):

Supplemental Video S5 (related to Figure 4):

Supplemental Video S6 (related to Figure 4):
