# Supplementary material for: Cholinergic activity is essential for maintaining the anterograde transport of Choline Acetyltransferase in Drosophila
Source: Sci Rep. 2018 May 23;8:8028. doi: 10.1038/s41598-018-26176-z (PMC5966444; doi:10.1038/s41598-018-26176-z)
Supplement: Supplementary file 1 — Supplemental Figures and Legends [file 41598_2018_26176_MOESM1_ESM.docx]

**Cholinergic activity is essential for the anterograde transport of Choline Acetyltransferase in *Drosophila***

**Swagata Dey^1, 2^, and Krishanu Ray^1, 2, 3^**

^1^ Department of Biological Sciences, Tata Institute of Fundamental Research, Mumbai, India.

**Correspondence:** Phone: +91-22-22782730, Fax: +91-22-22804610

E-mail: [swagatad86@gmail.com](mailto:swagatad86@gmail.com), [krishanu@tifr.res.in](mailto:krishanu@tifr.res.in), [krishanu64@gmail.com](mailto:krishanu64@gmail.com)

**^2^- corresponding authors, ^3^- lead correspondence**

**Table.S1: Details of the constructs used in the study.**

| ***Genotype*** | **Source** | **Description** |
| --- | --- | --- |
| *chaGal4>UAS GFP-ChAT/+* | Sadananda et al., 2012 | Expressing GFP tagged Choline Acetyltransferase (ChAT) |
| *cha^ts2^* | Bloomington 51627 | Temperature sensitive mutant allele of ChAT with loss of ChAT function, paralysis, and lethality manifesting at nonpermissive temperatures. |
| *chaGal4>UAS GFP-ChAT/+ ; cha^ts2^* | CG12345 (*ChAT*) | Expressing GFP tagged Choline Acetyltransferase (ChAT) in the background of homozygous *cha^ts2^* mutant. |
| *chaGal4>UAS KLP64D-GFP/+* | Sadananda et al., 2012 | Expressing GFP-tagged KLP64D subunit of Kinesin-2 |
| *chaGal4>UAS KLP64DΔT-GFP/+* | Sadanadna et al., 2012 | Expressing GFP-tagged tail-less (with cargo binding domain truncated) KLP64D subunit of Kinesin-2 |
| *chaGal4>UAS KLP68D-YFP/+* | Sadananda et al., 2012 | Expressing YFP tagged KLP68D subunit of Kinesin-2 in the cholinergic neurons |
| *chaGal4>UAS TQ /chaGal4>UAS sYFP* | Generated by A. Kulkarni K.Ray lab | Expressing Turquoise (TQ) and YFP under UAS in the cholinergic neurons |
| *chaGal4>UAS TQ-ChAT / +* | Cloned and generated for this study (K.Ray lab) | Transgenic with TQ tagged ChAT expression under UAS control |
| *chaGal4>UAS TQ-ChAT / chaGal4>UAS KLP68D-YFP* | Cloned and generated for this study (K.Ray lab)  CG12345 (*ChAT*)  CG7293 (*Klp68D*) | Expressing TQ tagged ChAT along with YFP tagged KLP68D under UAS control |
| *chaGal4>UAS GFP-ChAT/+ ; Klp64D^A8.n123^/Klp64D^k1^* | CG12345 (*ChAT*)  FBal0060615  FBal0102965 | Expression of GFP tagged ChAT in the background of null mutant (*Klp64D^k1^*, truncated at 13^th^ amino acid) and excision deletion mutant of Klp64D (*Klp64D^A8.n123^*) created by P-element insertion-excision. |
| *chaGal4>UAS GFP-ChAT/+ ; Klp64D^k5^* | CG12345 (*ChAT*)  FBal0102963 | Expression of GFP tagged ChAT in the background of homozygous *Klp64D^k5^* mutant |
| *chaGal4>UAS GFP-ChAT/UAS-KLP64D TEV-H; Klp64D^k5^* | CG12345 (*ChAT*)  CG10642 (*Klp64D*)  FBal0102963 | Full-length h of the *Klp64D^k5^* mutant by expression of *Klp64D-TEV-His* transgene in the cholinergic neurons by *chaGal4*. |

**Supplemental Movie Legends:**

**Supplemental Video S1:** **Rescue of homozygous *cha* mutant phenotypes by the expression of GFP-ChAT in cholinergic neurons.**

Climbing assay of the homozygous *cha^ts2^* mutant (N-3, n>10) and rescued (*chaGAL4>UAS-GFP-ChAT/+, cha^ts2^*, N=3, n>10) flies after rearing at 32°C for 24 hours. Both fly stocks were grown at 18^o^C until the adults emerged and then transferred to a water bath set at 32°C. The level of ChAT antigen was reported to reduce significantly in the homozygous *cha^ts2^* background after 24 hours at the non-permissive temperature, causing paralysis. We found that all homozygous *cha^ts2^* mutant flies remained at the bottom of the tube throughout the observation period. The assay was repeated three times with each batch of flies, and the result was 100%. The paralysis was rescued completely (100% flies) by the expression of the recombinant *UAS-GFP-ChAT* using the *chaGal4* driver. The video shows the movement of the flies transferred to a graduated vertical glass tube for a minute with a frame rate of 30hz. The homozygous *cha^ts2^* mutant adults were paralyzed and unable to climb whereas the *w, chaGal4>UAS-GFP-ChAT; cha^ts2^* (rescued) rapidly climbed up the tube within the period.

**Supplemental Video S2 (related to Figure 1):** **Developmental pattern of GFP-ChAT flow in the axons.** FRAP of GFP-ChAT in the axon segments of wild-type (*chaGal4>UAS-GFP-ChAT/+*) *lch5* neurons aged up to 76-79h AEL depicts temporally regulated anterograde flow at 78h and 79h AEL in a pseudocolor scheme. The frame rate of 10hz.

**Supplemental Video S3 (related to Figure 2):** **Developmental pattern of KLP64D-GFP flow in the axons.** Pseudocolored images of FRAP of KLP64D-GFP in the axon segments of wild-type (*chaGal4>uas-KLP64D-GFP/+*) *lch5* neurons aged as above depicts decreased flow at 77h AEL and subsequent increase at 78h and 79h AEL. The frame rate of 10 Hz.

**Supplemental Video S4 (related to Figure 3):** **Developmental pattern of KLP68D-YFP flow in the axons.** Pseudocolored time-lapse images of donor emission and sensitized FRET emission of TQ-ChAT and KLP68D-YFP coexpressed in the lch5 neurons at 78h AEL. The frame rate of 5 Hz.

**Supplemental Video S5 (related to Figure 4): GFP-ChAT flow in the axons after the hemicholinium treatment.** FRAP of GFP-ChAT in the axon segments of wild-type (*chaGal4>uas-GFP-ChAT/+*) *lch5* neurons aged up to 76-79h AEL in the presence of 100 µM HC3 depicts a lack of temporally regulated anterograde flow at 78h, and 79h AEL represented in a pseudocolor scheme. The frame rate of 10hz.

**Supplemental Video S6 (related to Figure 4):** **GFP-ChAT flow in the axons after the α-Bungarotoxin treatment.** FRAP of GFP-ChAT in the axon segments of wild-type (*chaGal4>uas-GFP-ChAT/+*) *lch5* neurons aged up to 76-79h AEL in the presence of 125 nM BTX represented in a pseudocolor scheme. The frame rate of 10hz.

**Supplemental Figures**


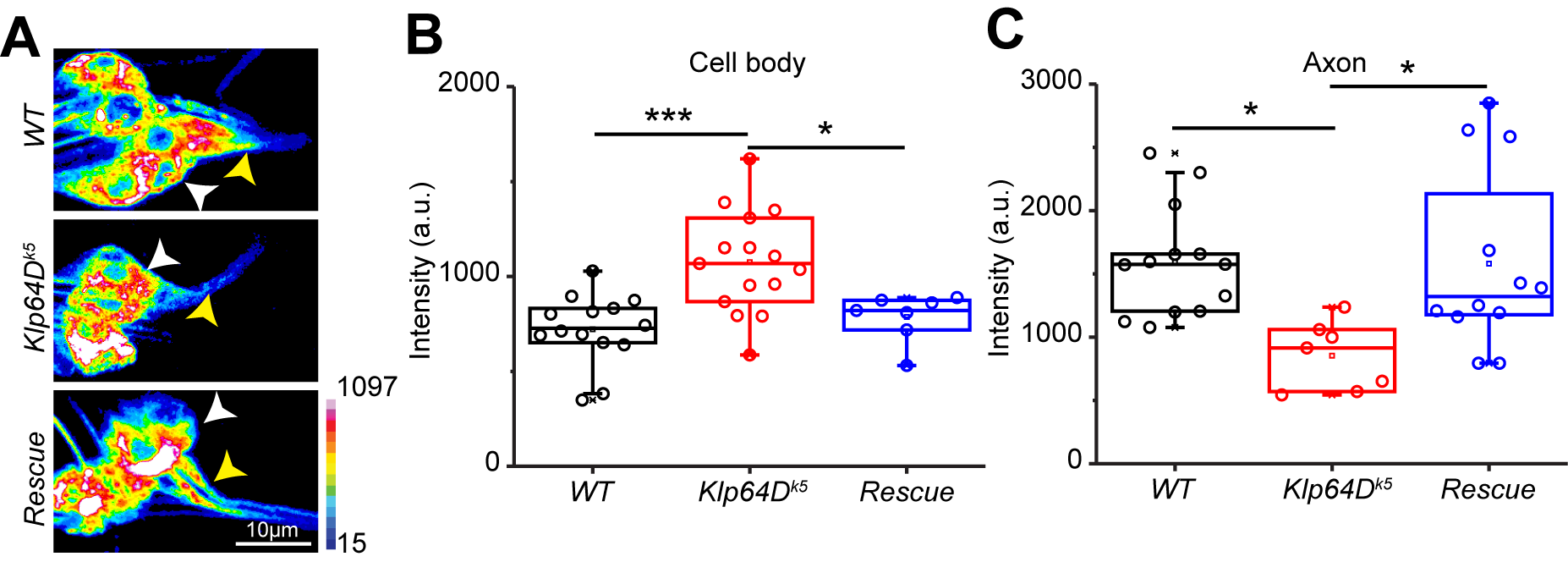


**Figure S1 (related to Figure 1). Axonal GFP-ChAT in Klp64D mutant and full-length rescues.**

(A) Pseudocolored images indicate levels of GFP-ChAT localizations in the lch5 neurons from the wild-type, homozygous *Klp64D^k5^* mutant, and rescue (*w, chaGal4>UAS-KLP64D-TevHis/+; Klp64D^k5^*) larvae. White arrowheads point the cell bodies, and yellow arrowheads mark the axons. (B-C) Intensities of GFP-ChAT measured in the cell bodies (B) and the axons (C) of lch5 neurons of the wild-type, homozygous *Klp64D^k5^* mutant, and full-length rescue larvae.

**Figure S2 (related to Figure 2). Transport parameters of Kinesin-2 subunits in *lch5* axons.**


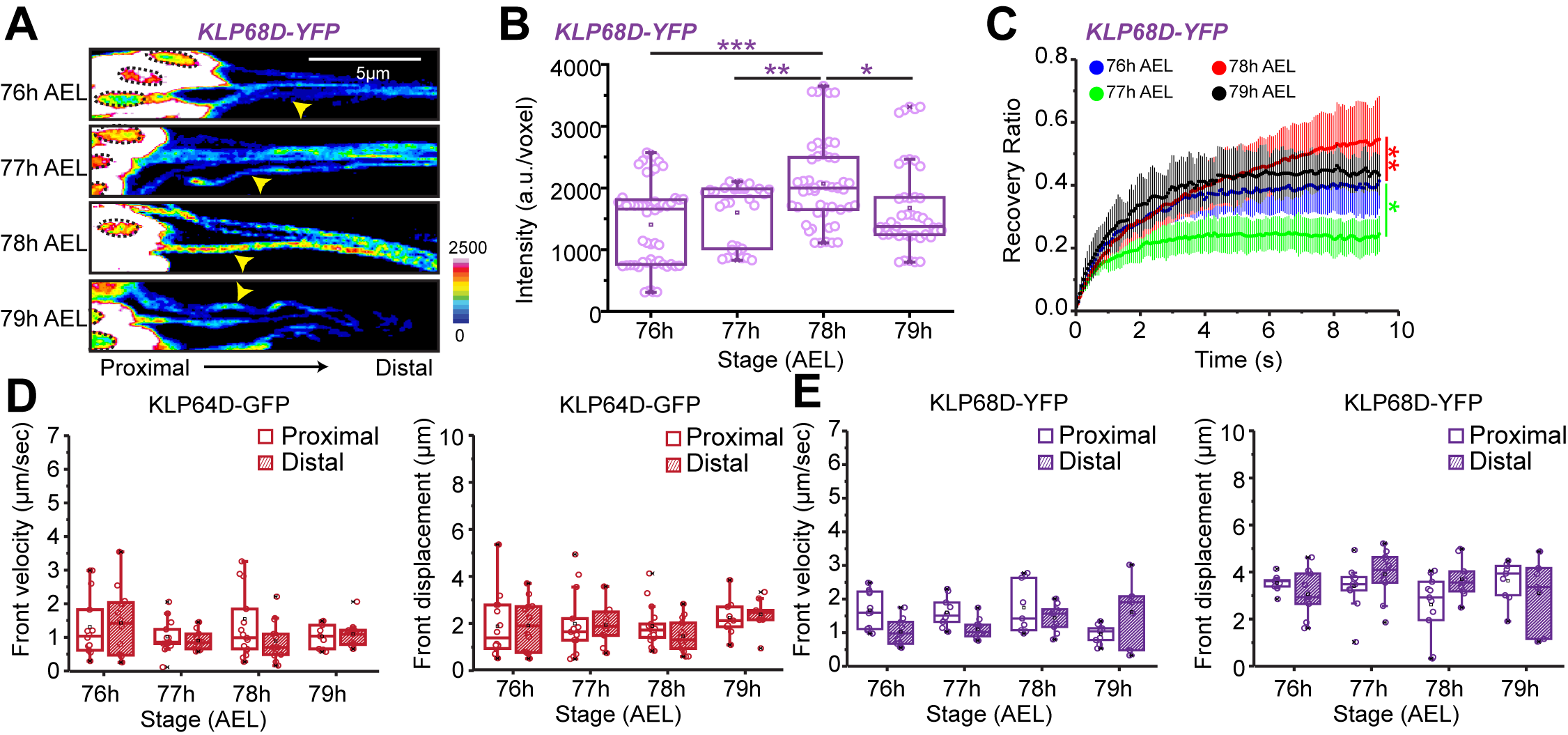


(A-C) Axonal accumulation and flow characteristics of KLP68D. Images presented in pseudocolored intensity heat-map depicts KLP68D-YFP localization in the *lch5* axons (A). The box plots show accumulation of average fluorescence intensity in the axon segments during 76-79h AEL (B). The line plots show relative fluorescence recovery (Mean + S.D.) of KLP68D-YFP in the same axon segment during 76-78h AEL recorded at 10 Hz.

(D-E) Box and scatter plots indicate anterograde and retrograde front velocities, and front displacements of KLP64D-GFP (D) and KLP68D-YFP (E), assessed from the respective kymographs.

The pairwise significance of differences was estimated using one-way ANOVA and the p-values (ns, * <0.05, ** <0.01, *** <0.001) are indicated on the panels.


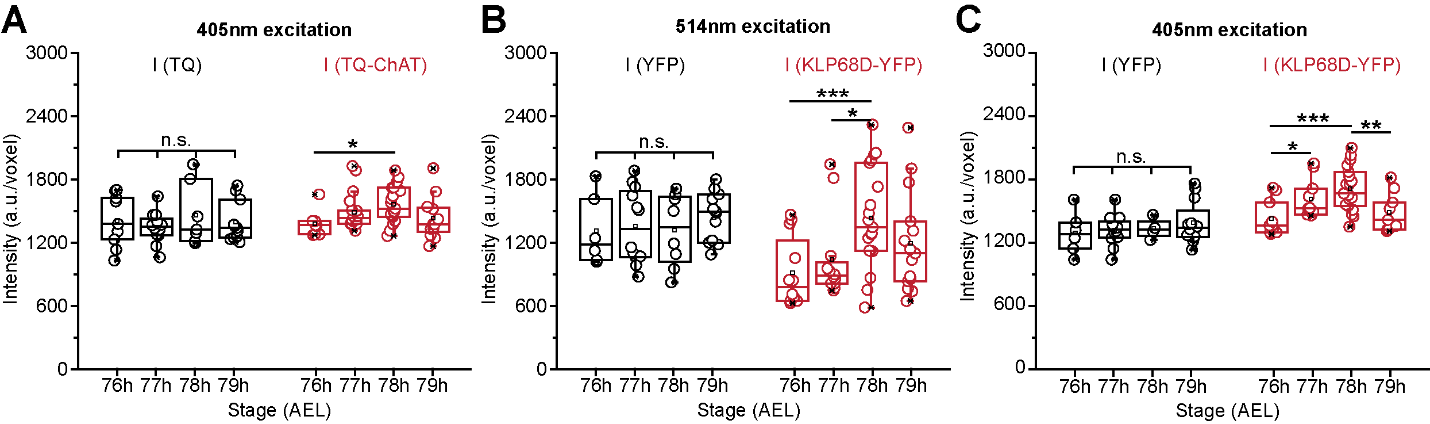


Figure S3 (related to Figure 3). Comparison of donor and acceptor intensities for TQ/YFP and TQ-ChAT/KLP68D-YFP pairs.

(A) Box plots depict average fluorescence intensities of the donor (TQ) in the axon segments from *chaGal4>UAS-TQ/UAS-sYFP* and *chaGal4>UAS-TQ-ChAT/UAS-KLP68D-YFP* backgrounds. The fluorophore was excited at 405 nm, and emissions were recorded at 490/10 nm.

(B) Similar plots depict the levels of the acceptors (sYFP or KLP68D-YFP) in the same axonal segments obtained at 514 nm excitation and 550/30 nm emission.

(C) Box plots depict sFRET emissions recorded at 405 nm excitation and 550/30 emissions from the same axon segments as A and B.

The pairwise significance of differences was estimated using one-way ANOVA and the p-values (ns, * <0.05, ** <0.01, *** <0.001) are indicated on the panels.


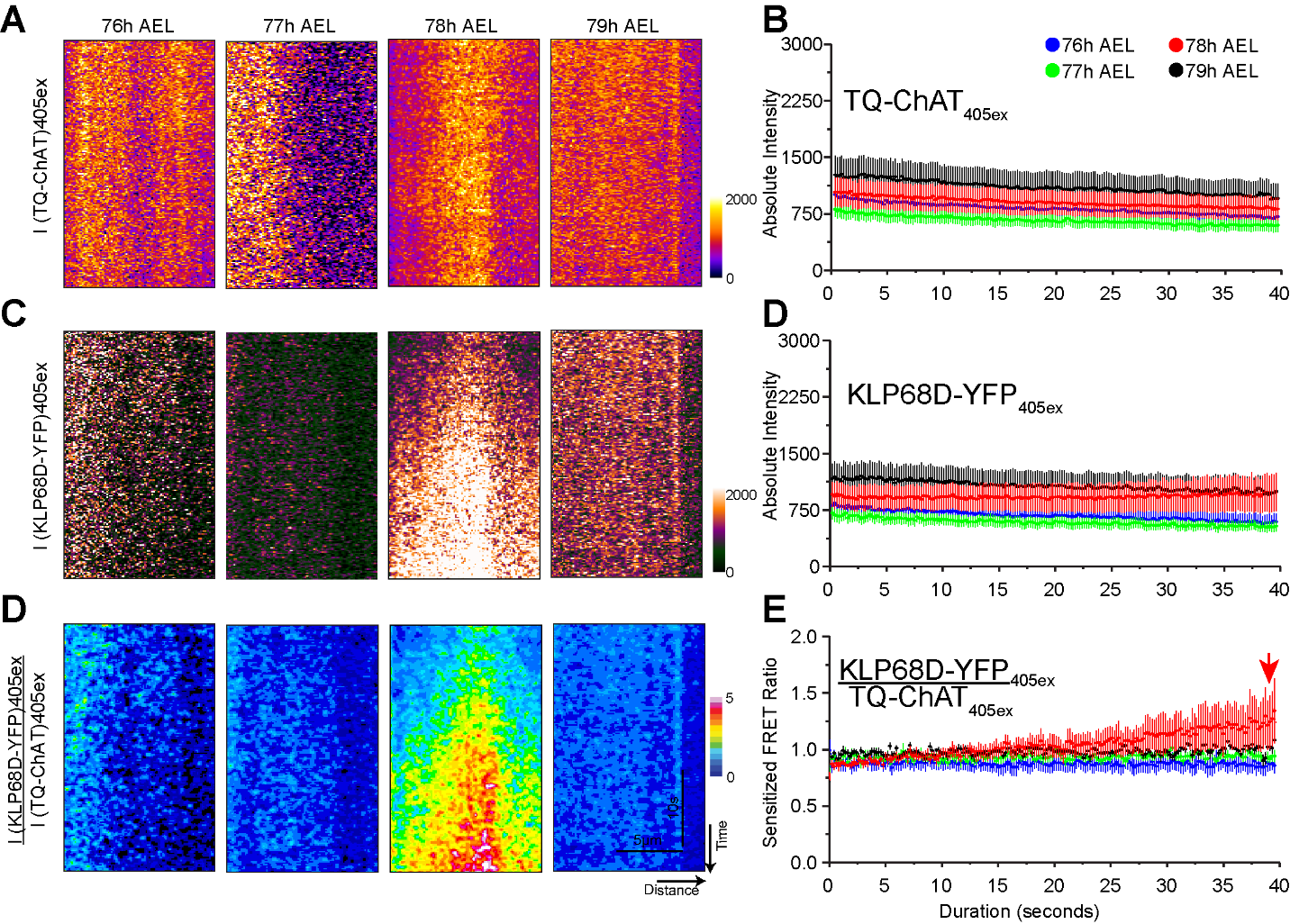
Figure S4 (related to Figure 3). Time-lapse observation of FRET between TQ-ChAT and KLP68D-YFP at 76-79h AEL.

Kymographs and associated intensity profiles depict the evolution of TQ and FRET emission, and corresponding sFRET ratios along the length of a typical axon, as recorded over an extended duration at different stages. Note the gradual increase in the ratio FRET with time (N≥8).


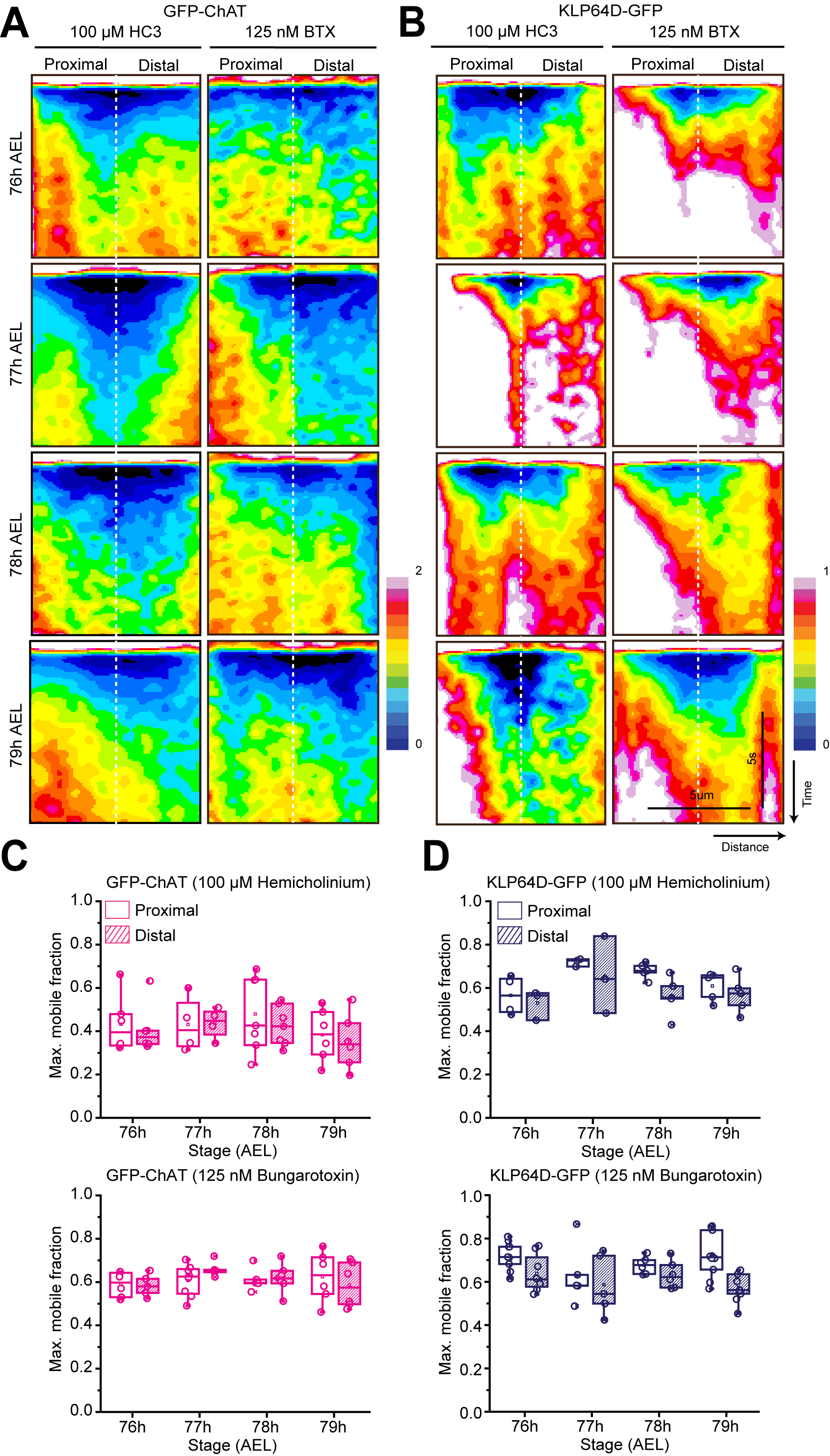


**Figure S5 (related to Figure 4). FRAP profiles of GFP-ChAT and KLP64D-GFP in lch5 axons upon HC3 treatment.**

(A-B) Kymographs represent FRAP profile of GFP-ChAT (A) and KLP64D-GFP (B) during 76-79h AEL in HC3 and BTX treated axons, in pseudocolor.

(C-D) Mean ± S.D. of maximum mobile fractions of GFP-ChAT (C) and KLP64D-GFP (D) during the period of 76-79h AEL in the proximal and distal segments of the FRAP region in HC3 and BTX treated axons. The pair-wise significance of differences assessed using ANOVA and p-values were not significant (>0.05).
